# Dysentery; Its Pathology, Causes, Etc., with Cases

**Published:** 1860-09

**Authors:** H. P. Ayres

**Affiliations:** Fort Wayne, Indiana


### Original Communications.

ART. I.—*Dysentery; its Pathology, Causes, and Treatment, with Cases.* By H. P. AYRES, M.D., of Fort Wayne, Indiana.

IT appears almost presumptuous to publish any article on the subject of dysentery, after it has elicited and received so much consideration from such men as Cheyne, Twining, Cornel, McIntosh, Stokes, Watson, and many other minds in this and other countries.

Studious investigation and patient research have characterized the interest of the profession in this prolific subject. Years of patient labor have been insufficient to satisfy the inquiries respecting its pathology and treatment. Ever varying in its phases, it has exhausted the resources of the materia medica to meet its mutations, and sometimes bewilders the profession to make applications to relieve the sufferings of the afflicted. At one time it is mild, gentle, and perfectly manageable; at another time, it sweeps a neighborhood or town with a blighting desolation, leaving in its march mourning households. Age, sex, time, and place are oftentimes put at defiance. The weak, the strong, the well fed and the poorly fed, fall victims to its attacks. The torrid, temperate, and frigid zones are visited by its ravages; and of dysentery it may be said, where man is found, there will some of its forms appear. The special pathology of dysentery, with its anatomical lesions, have long been settled; the colon and rectum being, without doubt, the points where the greatest derangement of the economy occurs, yet it may extend by sympathy or positive inflammation from the mouth to the anus, involving all the glandular and peritoneal textures of the abdomen. But where and from what causes the disease originates, by what process it is generated, how the poison is introduced into the system, the changes it produces in the organs of respiration and nutrition, how it develops itself in the colon and rectum, and why it assumes its various phases in different years, are questions yet open to investigation.

Why, during one year, is it mild, making its attacks upon all in the same manner, however differently situated in respect to houses, clothing, and diet—running just such a course, and yielding to a similarity of treatment? Why, during another year, does it assume a chronic, typhoid, or inflammatory type, having characteristics which mark it in every patient, not alone in the simple phenomena of the disease, but in its effects upon the eye, the lips, the tongue, the nose, the voice, and the skin? So marked are these phenomena that the physician recognizes the nature of the disease in a new patient from the physiognomy alone. We naturally trace results back to ascertain their first causes, but in no instance where the organism is perfect will disease be found; and in no case will disease or death invade the economy until the full period of life is run, unless secret results be superinduced by external matter, in the shape of aërial or ingestive poisons—the latter being so either from their own chemical qualities or becoming so from a morbid digestion.

Perceptible and present causes are sought to account for all diseases; and generally we seek for them in our food or drink, in heat, cold, seclusion, or exposure. But if heat, cold, food, or exposure are productive of the phenomena in diseases, why do they not produce such results whenever they are brought to act upon the body? Why have we dysentery of one type in the spring or summer, and a fac-simile in the autumnal months, or like phases of this disease in the extremes of temperature; or why do not similar productive causes, such as diet, clothing, drink, and locality, uniformly produce similar results?

If we attribute sporadic, endemic, or epidemic dysentery to either food, miasm, or locality, why do they not have, upon the various constitutions attacked, an effect corresponding to the strength and vigor of such constitutions? And yet such is not the case; we have seen the strong fall before the march of dysentery as rapidly as the weak, and the weak recuperating as rapidly from its attacks as the strongest: such facts have come to the observation of every practitioner. Now, if such results are true, we can come to no other conclusion than that there must be some agent or agents acting, whose phenomena and results are similar where-ever they are exercised. We say there is a *vis medicatrix naturæ;* but this power does not exist perceptibly when tested by arsenic and strychnine, and we think the same principle true in dysentery; when the poison producing it finds its way into the system, the results are as surely developed as are the effects of mineral or vegetable poisons, but of course in the same ratio as to the amount taken.

Strychnine or arsenic will produce their results in the torrid, frigid, or temperate zones; ergot will effect the strong and the weak in a similar manner; veratrum viride will produce like results on a thousand different patients, however circumstanced in relation to locality, food, water, or other causes. So is it in dysentery; there must be an agent generated or introduced into the system, possibly arising from a multiplicity of external causes, yet, whenever and wherever it is generated, a part or all of the phenomena of dysentery will appear.

Every physician knows that affections of the liver, spleen, stomach, gall-bladder, and other organs of the body, produce a series of phenomena peculiar to themselves, which are always present in the same ratio as the disease is mild or intense; and such results may as surely be looked for as may the effects of arsenic or any other poison.

If such results come under our observation in reference to diseases of the liver, stomach, or gall-bladder, why may not the series of phenomena in dysentery be traced to a previous affection of some of the functions of the economy?

The characteristics of dysentery are the same in all countries and in all latitudes, and there must be similar causes producing such a uniformity in results. Dysentery is but the manifestation of another disease, but in point of treatment it is a distinct local inflammation. The primitive diseased part is the blood, which may have been changed from its normal condition by animal, mineral, aërial, or vegetable poisons, received through the skin, lungs, or stomach; but whenever and however it originates, it, by the law of election belonging to some diseases and medicines, invariably manifests itself in the colon and rectum. Or the disease may be perpetuated by the poisonous secretion or excretion of the various organs along the alimentary canal, which have been impregnated with the poison as the blood circulated through their textures, thus frequently disappointing the expectations of the physician by a recurrence of the symptoms of dysentery after he thought a cure had been effected.

The elective power of medicines and diseases cannot be denied. Ergot, strychnine, and veratrum viride will affect the womb, nerves, and heart; the venereal virus will affect the throat; certain conditions of the stomach will affect the conjunctiva, motor nerves, or skin; certain forms of porrigo will only affect the scalp. So is it in dysentery—this peculiar state of the blood chooses only to affect the colon and rectum.

Neither the existence of indigestible food nor the indigestion of digestible aliment in the alimentary canal are in themselves necessarily productive of dysentery. Every practitioner of experience has observed children, during the prevalence of dysentery, using unripe fruit, and other substances equally pernicious, with the utmost impunity, and yet entirely escape the disease. We also, in lienteric states of the bowels, observe food passing, with no other change than a simple rounding of the corners, which is more the result of friction than digestion, yet without any appearance of the form of dysentery, of which we are now treating.

The same principle holds true during the prevalence of Asiatic cholera. Many persons, with the utmost impunity, use articles of diet proscribed by universal consent, and yet escape this dreaded scourge; while others, after the most rigid care, are its first victims. Such results cannot be attributed alone to an affection of the mind acting upon the functions of the economy, for the disease may be observed in children, where a fear of it does not exist.

We frequently find parents consulting the profession respecting the best means of preventing dysentery during its prevalence; and yet these very families are perhaps the greatest sufferers, while those who are perfectly indifferent entirely escape. We do not assume the position that care is no safeguard to health, but we do say, that in some diseases where the producing poison enters the body, the results are positive and certain, regardless of the most watchful care.

We have long been of the opinion, that if a series of observations, by which the proportionate amount of gases composing the atmosphere, during the prevalence of any epidemic, could be registered, with an analysis of the discharges from the bowels, we would find an exact ratio existing between them and the mildness or severity of the disease. We do not embrace the theory of Kircher, Holland, or Henle, respecting animalculæ being the origin and cause of disease. We think there are certain legitimate laws regulating the organism in health; and when there is an infringement upon these laws by extraneous matter, derangement in some or all of the functions of the body is an inevitable result.

All persons know that an excess of oxygen immediately affects the character of the red globules of the blood; while an insufficient amount equally affects them, but in a more deleterious manner. The blood may be diseased by an introduction of foreign substances through the skin, lungs, or stomach, or from a want of a proper elimination of substances generated in it. "Carbonic acid, urea and lithic acids, cholesterin and other elements of bile, and other matters which it is the office of the excreting organs to remove, may accumulate in the blood, and may become fertile sources of disease by their injurious presence."*

We also know that peculiar states of the atmosphere will affect the

---

* Carpenter's Physiology, p. 534.

fibrin of the blood, thereby producing an inflammatory state of the system; while the poisonous atmosphere surrounding a typhous patient has a contrary effect. Pathology abounds with facts which will sustain the position, that in all cases of dysentery there is a primitive diseased state of the blood, which, whenever circumstances develop the poison, either through the lungs, skin, or food, the manifestation of such poison in the blood is shown upon the colon or rectum, or both, making dysentery but a sequence of another disease.

The treatment of dysentery has been no less surrounded with difficulties than has its general pathology. Were I to select any disease, by which to prove that nature exerted a greater influence in the cure of disease than art, I would select the one under consideration; for, in its treatment, there have been the most antagonistical views respecting almost every article in the materia medica in reference to its curative powers. The remedy which, in the hands of one man, has acted as a specific, has been utterly and hopelessly abandoned by another. Remedies which have proved highly successful in one year, have proved inefficient during the succeeding season.

The data, from observation, respecting the causes and treatment of dysentery, differ so widely in conclusions, that the present treatment is largely of an experimental character. After making full allowance for climate, constitutional differences, and complications with other diseases, there is an identity in dysentery, wherever found, which we think should lead to a more successful and uniform method of treatment; but, until investigation reveals to us a fuller understanding of its pathology, we must content ourselves with our present remedies and appliances.

Among the remedies which have been most extensively used, mercury stands prominent. Physicians have used it in the East, the West, the North, and South, and the experience with it has been almost as diverse as the poles themselves.

Dr. Coley used calomel and opium in inflammatory dysentery, with success. Dr. Lewins and Dr. Hayns, of Java, gave scruple doses, and repeated the medicine, until the cure was effected. Dr. Hare, of India, had an unbounded confidence in the sulphate of quinine and injections, but remarks: "If that treatment will not answer, the profession must go back to a mercurial treatment, involving salivation; for there had been a decided increase in the mortality of dysentery since its abandonment." Dr. Papillaud, of Brazil, rejects calomel and uses sodæ sulph., calling it the *par excellence* among all remedies, and at the same time rejects all vegetable and mineral astringents. Dr. Baily relies on calomel, bleeding, and opium; his favorite prescription was: Calomel grs. ij, opii. pulv. gr. $\frac{1}{3}$, every three hours, until the gums were tender. Dr. Annesley recommends calomel in scruple doses, but warns the profession against producing the constitutional effects of the medicine. Dr. Cheyne, of Dublin, uses venesection, with calomel and opium, successfully, but doubts the propriety of salivation. Dr. Brown, of Glasgow, found venesection, calomel, and opium unsuccessful. Dr. Johnston, in his wide field of observation, relied upon calomel alone. Dr. McFarlane recommended from fifteen to twenty grains of calomel, with one of opium, as the first dose, and then two grains every six hours, until salivation was produced. Dr. Abercrombie, a man of wide experience, has but little confidence in calomel; but "in some cases" recommends one grain of calomel, with pulv. Doveri, every four or five hours. Dr. Turnbull, of Liverpool, recommends a pill composed of four grains of calomel and one of opium, as the first dose, to be followed by smaller ones until the gums are sore. Dr. Twining, whose experience is unsurpassed, says he has seen salivation without curing dysentery; and, while he does not reject calomel, highly recommends an ipecacuanha treatment. Dr. Bell gives calomel in doses of fifteen or twenty grains at once, or five grains of pure calomel every three hours, until the bowels are emptied, but remarks: "If this course does not soon effect a cure, it should be abandoned." Dr. Christison relies on opium and sulphate of quinine. Dr. J. T. Garrison, in his report to the American Medical Association, says he found calomel applicable in almost every case; and with it, ipecac., sulphate of quinine, and opium was very successful. Dr. Wyman, of Cambridge, Mass., manifests but little confidence in calomel, but relies upon purges, followed by opium. Dr. Condie uses calomel in one-grain doses every two or three hours. Dr. Dunglison does not advocate the use of this remedy warmly, and rejects the idea that salivation is a specific; he recommends its careful use. Dr. Eberly warmly advocates its use, even to ptyalism. Dr. Stokes remarks: "Thus, when you have given calomel for twenty-four or forty-eight hours, and find no signs of salivation appearing, you should be cautious how you proceed." Dr. Good advocates the use of mercury in dysentery. The English army surgeons found calomel good in dysentery in India, while, during the same year, the French army in Egypt found it decidedly pernicious.

The lists might be greatly lengthened, to show the diverse opinions which exist among the best men in the profession respecting the use of a single drug in dysentery. We need not think it strange, when the leading minds in the practice of medicine differ so widely in their views, that the profession is yet without any fixed principle in the treatment of this disease.

We are aware the disease in the torrid and temperate zones is largely influenced by the different circumstances under which it exists; and did we find the discrepancy existing alone in different latitudes, the case would naturally be changed. But authors and practitioners in the same latitudes, or even in the same city, widely differ not only in remedies, but in the divisions or phases of the disease itself; some having one form, others having two, three, and four divisions, leaving the matter as unsettled as are the remedial measures.

But the discrepancy in reference to calomel in the treatment of dysentery is only one among fifty other drugs, which are claimed by some as efficient or specific remedies. Venesection, ipecac., sulphate of magnesia, sulphate of quinine, acetate of lead, acetate of morphia, opium, blue mass, Dover's powder, muriatic and nitric acids, catechu, kino, copaiba, tannic acid, cold water injections, white oak, prepared chalk, cascarilla, cinchona, and a host of counter-irritants, all of which it is proper for the physician to use, if he can but establish the point that he is treating dysentery. But while we would complain of the indefiniteness in the division and treatment of this malady, we would avoid the labyrinthic divisions which characterize our cutaneous nomenclature. Simplicity in the classification and treatment of disease is as absolutely necessary to success, as it is in the construction of machinery to its success and durability. The complicated and useless combinations which characterize old formulas have rapidly given way to a more reasonable, simple, and efficient practice; and the reformation thus begun will continue, until the practice of medicine will be stripped of much of its present mysticism.

Dysentery always appears in one of two forms: inflammatory or acute, and subacute. Hence we think the divisions of acute and subacute quite sufficient, and better calculated as a basis upon which to found a treatment than the variety of names and subdivisions mentioned by different writers. The acute form may become subacute; and conversely, the subacute may become acute; but the chronic and typhoid states, which we frequently see, may result in either form of attack, but should only be looked upon as results.

In the acute form, we may have a febrile state of the whole body from the first onset of the disease; or we may have a cool skin, with a rapid pulse, and great thirst; or we may have a burning skin, and no very marked acceleration of the pulse. We may have tenderness of the bowels, with intense pain in the head, or we may have one of these phenomena without the other; but always in this form, restlessness, and loss of appetite. In the subacute variety, we may have all the phenomena of dysentery upon the colon and rectum, with very little pain either in the abdomen or head; the patient sometimes appearing entirely free from any unpleasant sensations unless from tenesmus. The appetite may remain unimpaired, and no increase of thirst be present. Yet from both these states have we, as before intimated, seen the chronic and typhoid types arise. The same reasoning is true in reference to other complications. A morbid state of the stomach, engorged liver or spleen, may be complicated with the disease, but only exist as complications, and should not give rise to such divisions as intermitting and tertian dysenteries. If this disease were always treated *pro re nata*, and without reference to specifics, much of the discrepancy in treatment would cease, and there would be a more uniform practice established. Acute dysentery should be treated as an inflammatory affection, without reference to any previous experience, as this inflamed state may, with every year and case, have new phases and types. In the subacute form, some astringent, a dose of calomel, of salts or oil, may be sufficient to check the dysentery; while the very same remedies, in the acute form, might only increase the inflamed, congested, and irritated state of the lower bowel. I am satisfied these two states may exist in epidemic, endemic, and sporadic dysentery, yet in all cases involving the same principle in the treatment.

During the year 1845, dysentery prevailed in Northern Indiana as an epidemic, and assumed a decidedly inflammatory character. The stomach, the whole alimentary canal, and more especially the liver, were involved in the trouble. There was great tenderness of the bowels, and in many cases severe tympanites, with an excruciating pain in the anterior portion of the brain. For one or two days there was a slight disturbance of the bowels, exciting but little attention. At the close of the second day there would be a free serous discharge, succeeded in a few hours by a sanguineo-mucous, and then by a sanguineo-purulent one, great nervous sensibility, restlessness, rapid emaciation, cadaverous features, sunken eyes, and sometimes aberration of intellect. The pulse was full and hard at the commencement of the disease, with an increase of about thirty beats over its natural number. The tongue was dry, the thirst insatiable, and fluids, when taken, were frequently rejected from the stomach as soon as swallowed. The whole organism appeared to be involved in a general inflammatory action, which, when unrestrained, soon brought on disorganization and dissolution. I have noted several cases as they occurred in my own and other physicians' practice, as illustrative of the facts in question.

CASE FIRST.—Miss E. S., aged twenty-three, was robust and fleshy, with ruddy cheeks, dark hair, and of a hopeful, cheerful disposition. The bowels were for two days affected with slight diarrhœa, causing no apprehension of any serious difficulty.

On the beginning of the third day the discharges rapidly assumed a muco-sanguinolent character, often passing clear blood, with severe and increasing tenesmus. The bowels became painful and tympanitic; the urine was voided frequently, but in small quantities. But the greatest pain was in the cerebrum, running from temple to temple, and spasmodic in character.

During the paroxysm the pain was most excruciating, causing the patient to cry out, notwithstanding her great fortitude. I took a sufficient quantity of blood from the arm to soften the pulse, which for the time being relieved the pain. The abdomen was rubbed with the following:—

℞.—Ol. olivæ,

Aquæ ammoniæ, āā ℥iss;

Ol. conii,

Ol. terebinth,

Tinc. camphoræ, āā ℥ss.

After the abdomen and back were freely rubbed with the liniment, several folds of light flannel were wrung out of warm hop decoction and applied to the bowels, and kept warm with a hot dinner-plate laid upon the flannel, and changed for another as it became cool. The closet-stool before being used had a handful of hops thrown into it, with two or three pints of hot water, the effect of which was very grateful to the patient, and salutary to the diseased parts. Dover's powder and ipecac. were administered every three hours, in sufficient doses to produce a slight nausea, and to some extent mitigate the pain, yet it did not entirely control the action of the bowels. To allay the thirst the patient was permitted to drink freely of mucilaginous water, and to take the following, to alternate with the Dover's powder and ipecac.:—

℞.—Spt. Mindereri, ℥ij;

Syr. ipecac. ℥i;

Tinc. veratri vir. ʒss.

M.—A dessert spoonful every three hours.

Sometimes I gave the latter every hour, without any other medicine, and succeeded in subduing the inflammation. But, notwithstanding this treatment, the violent pain in the head returned, and I could only give relief by again resorting to venesection. After this the trouble in the head did not return. When the severity of the inflammation was subdued, the following was ordered:—

℞.—Hydrarg. cum. cretâ, ℈iij;

Plumbi acet. ℈j;

M.—Ft. pulv. No. V.

One to be taken every three hours, until bilious discharges were produced. As is frequently the case in the treatment of dysentery, a diarrhœa succeeded, which was relieved by the following:—

℞.—Tinc. opii,

Tinc. kino,

Bals. copaibæ, āā ʒiij.

M.—Give six or ten drops to a child two years old; a teaspoonful to the patient every two hours until better.

The patient recovered under the use of an excellent article of port wine, tonics, and a generous diet, but with the entire loss of her hair, the latter fact characterizing many of the severe cases of this year.

CASE SECOND.—Miss H., aged nineteen. Nervo-bilious temperament; the latter largely predominating. Stature short, stout; black hair; ruddy skin; sprightly and cheerful in manner. The dysentery was ushered in with the same phenomena that characterized the preceding case, excepting the severe cerebral pain. The pain in the abdomen was severe, with great tenesmus, and high febrile action. The case was treated with calomel and opium; first a large dose, followed by successive smaller ones, combined with astringents, to check the action of the bowels. The result was, that the tenderness and tympanites were increased; all the symptoms were aggravated, and the patient died. The result of the two methods of treatment was, that the cases in which the treatment was first of an antiphlogistic character, without any special reference to the local disease, with but few exceptions recovered; while those who were subjected to the calomel and opium treatment died. The error in the above case was the want of an active antiphlogistic treatment, under which the patient might probably have recovered. Sulphate of magnesia was used in several cases, which terminated favorably, owing undoubtedly to its anti-febrile qualities.

CASE THIRD.—A child two years old. Commenced with a slight diarrhœa. Second day, sanguineo-mucous stools appeared. Evacuations and tenesmus were characteristic of severe dysentery. Calomel and Dover's powder were administered to procure bilious discharges; castor-oil was given to assist the operation. Injections of starch and laudanum were used to relieve the tormina, but were immediately and forcibly rejected. The calomel not procuring healthy operations, it was repeated, which only aggravated the morbid symptoms, and the patient finally died. In the rectum the rugæ appeared like rings, one above the other, red, and deeply congested; the abdomen and bowels were largely distended with gas.

We look upon the last case as a parallel one with the second, both illustrating the necessity of ascertaining at what point and in what manner the economy needs to be assisted. We think in this case the calomel should have been preceded by an antiphlogistic course.

CASE FOURTH.—John E., aged fifteen. A fine, healthy boy, living in easy circumstances, studious and precocious. The *Water Cure Journal* had been sent to the family, and then taken regularly. It contained an article on curing dysentery by cold water injections, in imitation of Dr. Twining.

His attack was a severe one. The cold water injections were assiduously given according to directions, which had the effect to check the bloody discharges from the bowels. The fourth day he became very weak and restless. Becoming alarmed on the sixth day, I was called in, and found the bowels were not troubling him severely, but there was a constant regurgitation of bile from the stomach into the mouth, requiring an effort, every few minutes, on the part of the patient, to free his mouth and throat to prevent suffocation. The skin was sallow or leaden, the eyes sunken, the nose pinched, and the countenance anxious. I gave him a full dose of hyd. cum cretâ, followed every half hour with a tablespoonful of water containing sup. carb. of soda. In two or three hours the regurgitation entirely ceased; the bowels passed healthy bilious matter, but the patient continued to grow weaker, notwithstanding every effort was made with tonics and stimulants to sustain the powers of life.

The water accomplished the reduction of the inflammation, which was very well; but it was used to such an excess that the lower bowel was paralyzed and its action suspended. The functions of the liver and other organs were entirely neglected, so that the system necessarily sank under such indiscretion. Had the lad used the water, and at the same time attended to the functions of the liver and bowels, he would in all probability have recovered. In all cases during this year, as the treatment approached an antiphlogistic one, success was the result; and as this principle was lost sight of, disappointment followed.

One of the strong characteristics of this epidemic was that the largest number of cases was among adults, and those generally females. When it attacked children it was more manageable, and the recovery was more rapid.

In 1854 we were again visited by dysentery, but it was entirely different from that of 1845, the grade of inflammation being subacute, and generally attacking children. Physicians resorted to the usual remedies, but did not meet with the success wished for; many of the remedies rather increased than removed the disease.

A similar epidemic broke out in a neighborhood eight or ten miles from town, but, owing to the distance, the citizens resorted to a home remedy, which was a decoction of white-oak bark. With this remedy they were entirely successful in their worst cases. Of course physicians profited by the suggestion. The treatment they adopted, was to evacuate the bowels with a mild cathartic, and then use astringents, which uniformly resulted in convalescence. But this treatment in the epidemic of 1845 would have been fatal, as it proved to be in some instances.

In 1856 we were again visited with the same disease. In this year there was another form of treatment required. The decided antiphlogistic course of 1845 was not only unnecessary, but unsuccessful; yet not less so than the astringent course pursued in 1854. Cathartics were absolutely necessary, and the patient rapidly convalesced under their judicious administration. The largest number attacked was among children, yet many adults suffered. Any derangement of the bowels, teething, and all indisposition appeared to terminate in dysentery. In this year, the calomel treatment was decidedly successful; and the majority of cases yielded to one or two doses, after which the patient would rapidly recover. A number of cases occurred, however, in which the patient neglected the matter, or resorted to some nostrum; in such cases the disease would speedily assume new phases, generally of an inflammatory type, which would require another treatment; and frequently such cases would assume a typhoid state, from which recovery was very slow.

Thus, in 1845 we treated dysentery strictly as an inflammatory disease, using venesection and other antiphlogistic remedies; in 1854, as a subacute, in which an evacuation of the alimentary canal and astringents constituted the successful treatment; in 1856, as a subacute kept up by a morbid state of the liver, spleen, and other organs, in which cathartics were absolutely necessary. In each of these years there were instances of chronic, typhoid, and ulcerative states of dysentery, which may form the subject of another paper. Here, we think, is the reason of the great discrepancy in the treatment of dysentery: the peculiar character or type has not been sufficiently illustrated in which calomel, venesection, ipecac., opium, and the various remedies which have been and are reputed as specifics, have been successful; and yet in the hands of others have entirely failed. It is true, Cullen, Sauvages, and Linnæus have not placed it among inflammatory diseases; but, with all deference to those who differ, dysentery should have the same place in our nomenclature as pleuritis, gastritis, or metritis. In each of these the proper treatment has long been settled by the profession, but with no better reasons than would apply to dysentery.

It is not necessary in all cases of pleuritis or metritis to bleed or give tartar emetic or calomel; we use such remedies according to the exigencies of the case, but we always keep in mind the inflammatory character of such diseases.

Just so do we think respecting dysentery. Place it among our inflammatory diseases, and the treatment will become as uniform as in pneumonia or pleuritis, which may or may not be complicated, like dysentery, with other diseases. I am aware many treat it as an inflammatory disease, and would repudiate any other treatment; but nevertheless the prevailing feeling with the profession is to have a specific in dysentery, when it can alone be successfully treated on general principles.

